# Spatial Arrangements of Connexin43 in Cancer Related Cells and Re-Arrangements under Treatment Conditions: Investigations on the Nano-Scale by Super-Resolution Localization Light Microscopy

**DOI:** 10.3390/cancers11030301

**Published:** 2019-03-04

**Authors:** Götz Pilarczyk, Franziska Papenfuß, Felix Bestvater, Michael Hausmann

**Affiliations:** 1 Kirchhoff-Institute for Physics, University of Heidelberg, Im Neuenheimer Feld 227, 69120 Heidelberg, Germany; goetz.pilarczyk@kip.uni-heidelberg.de (G.P.); f.papenfuss@gsi.de (F.P.); 2 Department of High Content Analysis of the Cell “HiCell”, BioQuant, University of Heidelberg, Im Neuenheimer Feld 267, 69120 Heidelberg, Germany; 3 Core Facility Unit Light Microscopy, German Cancer Research Center (DKFZ), Im Neuenheimer Feld 280, 69120 Heidelberg, Germany; f.bestvater@dkfz.de

**Keywords:** connexin43, breast cancer, single molecule localization microscopy, cancer treatment

## Abstract

Cancer studies suggest that the spatial localization of connexin43 (Cx43) could play an important role during tumor genesis and the formation of metastasis. Cx43 has been shown to be upregulated in cancer cells; thereby a shift from Cx43 normal localization in gap junctions in the cell membrane towards a primarily cytoplasmic localization was observed in many studies. So far neither the spatial arrangements of Cx43 in breast cancer cells nor the effects of treatment outcome (ionizing radiation and antibody therapy) on the spatial arrangements of Cx43, have been microscopically studied on the nanoscale. This has brought up the idea to study the micro- and nanoscaled spatial Cx43 arrangements in a model of breast cancer-related cell types, i.e., SkBr3 breast cancer cells, BJ fibroblasts, and primary human internal mammary artery endothelial cells (HIMAECs). The cells were treated with neuregulin1 (NRG1), trastuzumab (Herceptin), or 6MeV-photon irradiation at a dose of 4 Gy. NRG1 stimulates further NRG1 release in the tumor endothelium that may lead to an enhanced tumor protective effect whereas Herceptin, used in antibody treatment, works in an antagonistic fashion to NRG1. After fluorescent labelling with specific antibodies, the molecular positions of Cx43 in the perinuclear cytosol and in the cell periphery at the membrane were determined for the three treatment related applications (NRG1, trastuzumab, 4 Gy irradiation) using confocal laser scanning microscopy (CLSM) and single molecule localization microscopy (SMLM). These techniques enable investigations of Cx43 enrichment and topological arrangements of Cx43 molecules from the micro-scale of a whole cell to the nano-scale of single molecules. In SkBr3 cells with and without radiation treatment high density accumulations were detected which seem to be diluted after NRG1 and trastuzumab treatment although the SMLM distance frequency distributions did not significantly vary. In BJ fibroblasts and HIMAECs differences between periphery and perinuclear cytosol were observed after the different treatment processes. HIMAECs showed significant Cx43 accumulation after NRG1, trastuzumab, and radiation treatment in the perinuclear region whereas in the periphery radiation has less influence as compared to the control. BJ cells were reacting to the treatments by Cx43 accumulations in the perinuclear region but also in the periphery. In conclusion, it was shown that by using CLSM and super-resolution SMLM, treatment effects on the spatial and thus functional arrangements of Cx43 became detectable for investigations of tumor response mechanisms.

## 1. Introduction

Connexins are currently the subject of extensive debate about their action as key factors in the development, maintenance, and regulation of tumorigenesis in several forms of cancer progression and therapy [1,2,3]. In this context the interaction of connexin43 (Cx43) activity and the development of breast cancer has been reviewed [4,5,6]. The activities of different members of the connexin family can be classified into four main areas [7,8]. The first and second areas of activity relate to connexin transmembrane channel forming capacity which is a key factor for tumor suppressive access of chemotherapeutic drugs on one hand, but on the other hand, it is also a main pathway for intercellular communication in terms of metabolic optimization and balancing local tumorigenesis with local environment [9,10,11]. The intercellular communication is not restricted to cells of one type but happens also between different cell species. This is resulting in the possibility to control tumor development via mobile non-tumor cells and only slightly tumor associated cells [12,13]. Besides the modulation of cancer cells by non-cancer cells flanking by transiently, the opposite case, where breast cancer cells released from the primary tumor induce metastases can also be observed. Again Cx43 is involved [14]. The third area of connexin activity deals with the C-terminal tails facing the subcortical area of the cytosol and their accumulation of regulation sites being a target for kinases and phosphatases but acting also as membrane located starting points for cytosolic kinase signaling cascades [15,16]. The fourth area of connexin activity covers the intracellular located function of connexins as indicators and hubs for cytosolic regulation pathways [9,10,17]

Inside bulk tumors several cell types cooperate in the progression and maintenance of the entire tissue construct, the most prominent besides the particular tumor cells being fibroblasts and endothelial cells [18,19,20]. The artificial combination of the mentioned cell types is sufficient to reconstruct tumor spheroids with growth behavior and resistance and vulnerability, respectively, to therapeutic drugs comparable to naturally occurring tumors [21,22,23]. Besides such evidences from histopathology, tissue physiology and artificial organ approaches it has been reported that blood released protein factors like transthyretin (TTR) can act as reliable serum tumor markers. Its nature as a cytokine under control of signal transducer and activator of transcription, type 3 (STAT3) relates its occurrence closely with tumor progression and its targets being endothelial cells, epithelial cells, and alveolar cells where it stimulates tumor progression [24].

In breast cancer onset and progression, a transient decrease of Cx43 containing gap junctions is accompanied by a reorganization of the following generation of gap junctions. While non-malignant mammary epithelium develops gap junctions between epithelial cells and with supporting myoepithelial cells in mammary tumor tissue gap junctions between tumor cells and endothelial cells are the predominant location of Cx43 [25]. During the process of gap junction reorganization the amount of Cx43 protein not organized in gap junctions is increased [26]. The described dynamic dysregulation of Cx43 is under control of HER2 receptors (also called ErbB receptors), the typical cell proliferation modulators in breast cancer [27]. By the use of advanced co-culture experiments in networks of two-dimensional silicon based microfluidics, it can be demonstrated that tumor organization, gap junction establishment, and intercellular compound transfer are anisotropic and non-random and reflect tumor progression signal spreading along spatial routes decorated with Cx43 containing structures like hemi-channels, gap junctions, connexons and tunneling nanotubes [28]. In this context, the experimental finding that extracellularly applied Cx43 containing membrane rafts attenuate breast cancer cell aggression supports the assumption of role of Cx43 as a tumorigenic regulator [29].

It was shown by us that plasma membrane located Cx43 is with nucleograde characteristic co-transported with ErbB2 receptors after the application of external factors known to mobilize ErbB2 receptors. Such external factors were the generic ErbB2 stimulator peptide neuregulin-1β and radiation therapy mimicking doses of high energy irradiation [30]. Many factors make the Cx43 dynamics a favorite target for investigating mammary cancer development: These are for instance: (a) the function of connexin in several cancer related cell types, (b) the activity under terms of both tumor integrity and vulnerability, (c) the close functional, spatial and regulative association with main membrane receptors known to have key significance in breast cancer development.

Cx43 is one of the most prominent members of the 21 connexin iso-forms that have been identified in the human body [31]. Connexins are the primary components of gap junctions which form intercellular channels connecting neighboring cells. A gap junction channel is built up by two hemi-channels, the so called connexons which are connexin hexamers. Connexins are about 4 nm in size whereas the connexons are about 9 nm x 9 nm. Each connexin is organized in 4 trans-membrane α-helices connected by two extra-cellular and one intra-cellular loop. Since connexins only have a half-life of 1.5 to 5 h [32,33], this high turn-over rate is often associated with an accumulation of connexons in cytosolic plaques of 0.2–1.0 µm^2^ size [34] and pools to continuously replace and synthesize connexins.

Gap junctions regulate intercellular exchange of small molecules or inorganic ions, a process known as gap junction intercellular communication (GJIC). Since 1966 when Loewenstein et al. [35] for the first time published that cancer cells lose their ability of GJIC [36,37], connexins have become matter of interest in investigations of tumor genesis, metastasis formation [38,39,40] and the ability to restore GJIC for possibly gap junction mediated propagation of cell death signals amplifying chemotherapeutic effects [41,42,43,44]. Cx43 has been studied in a variety of cancers [45], e.g., prostate, lung, liver, brain, and especially breast [26,27,46].

Studies that investigate Cx43 expression and localization suggest a consensus that GJIC is lost during breast cancer development and most often an increased cytoplasmic appearance was observed [47,48]. This may be independent of Cx43 expression levels since increased or decreased Cx43 expression levels are not necessarily correlated with increased/decreased GJIC if the increase/decrease is due to cytoplasmic localization. However, drug sensitivity seems to be dependent on the formation of GJIC [46]. A potential role of Cx43 has not only been described in breast cancer genesis and metastasis development [39,40,41,49] but also in angiogenesis [50,51,52].

Based on these findings that suggest an important impact of the spatial organization Cx43 in the cell membrane and cellular cytosol for tumor and metastases developments as well as treatment response, the aim of the following investigations has been to analyze the spatial organization of single Cx43 molecules in tumor relevant cell model systems by means of confocal laser scanning microscopy (CLSM) in combination with single molecule localization microscopy (SMLM) [30,53,54] as a novel approach towards a better understanding of mechanisms behind non-junctional activities of Cx43 in tumor tissues. Recently, SMLM has successfully been used to analyze the spatial organization of membrane receptors in glioblastoma cell lines [55] or breast cancer cell lines [30,54]. SMLM has the advantage that it can be applied on cell surfaces but also inside 3D-conserved cells [56,57] or cell nuclei [54,58,59,60].

Here, SkBr3 cells were used as a drug-sensitive breast cancer cell model, Her2+ with reduced Cx43 expression but a tendency to form Cx43 plaques [27]. As a model for blood vessels relevant in angiogenesis, primary human internal mammary artery endothelial cells (HIMAECc) were used. Furthermore, the fibroblast cell line BJ was used as a model for connective tissue which could also appear in the tumor environment. These cell types were subjected to neuregulin1 (NRG1) stimulation, radiation or trastuzumab treatment and the spatial distribution of fluorescently tagged Cx43 molecules was analyzed in the cell periphery and the perinuclear cytosol in comparison to untreated cell controls.

For the following experiments, CLSM was applied to obtain a visual impression of the Cx43 distribution in the whole cell and to find out regions and patterns of Cx43 accumulation. For quantitative nanoscale analysis, an established embodiment of SMLM was applied that allows switching of fluorescent dyes between spectral “on” and “off” states [61,62]. This facilitates a spatial registration of the loci of single fluorescent molecules and thus spatial separation in the 10 nm regime. The fluorescent molecules randomly return from an induced dark state back to the emission state resulting in short bursts of fluorescence. The determination of the centre-of-mass (barycentre) of an Airy disc of a single molecule image approximates the location of the emitting molecule. This allows a precise localization of the object molecule and the calculation of molecule distances in the 10 nm regime [55,56,57]. Using the matrix of the coordinates of fluorescent antibodies labelling the Cx43 proteins, all acquired positions and distances can be analysed on the basis of Ripley’s point-to-point distance histograms and analysis procedures [63] before generation of an image. After data analysis artificial “pointillist”, super-resolution images are a graphical representative, in which results of Ripley’s data analysis can additionally be encoded.

## 2. Results

### 2.1. SkBr3 Breast Cancer Cells

**Figure 1:**
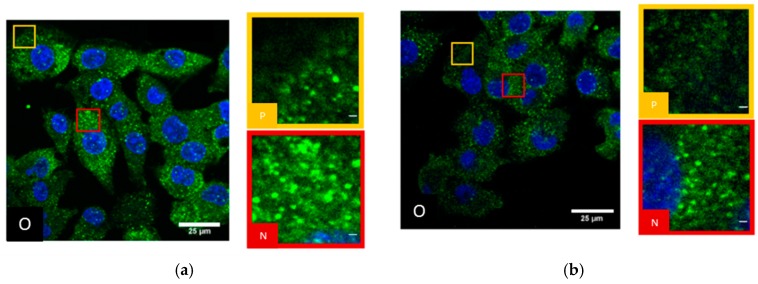
Confocal laser scanning microscopy (CLSM) images of SkBr3 breast cancer cells: (**a**) untreated; (**b**) after 4 Gy irradiation and 50 min recovery; blue: Cell nucleus after Hoechst34580 staining; green: Cx43 specific antibody labelling and Alexa488 staining. (O) Overview with selected peripheral and perinuclear regions. (P) Image section of the periphery of a SkBr3 cell; (N) image section of the perinuclear region of a SkBr3 cell. Scale bar: 1 µm.

SkBr3 cells did not show a significant difference between the cell periphery and the perinuclear cytosol. All over the cell Cx43 is distributed and forms accumulations (Figure 1a). However, at the perinuclear regions in many cells, the accumulations were more densely neighbored than in the periphery (Figure 1a). After irradiation with 4 Gy and 50 min recovery of the cells, the same pattern of Cx43 distribution was observed (Figure 1b) but the accumulations were less densely distributed.

**Figure 2:**
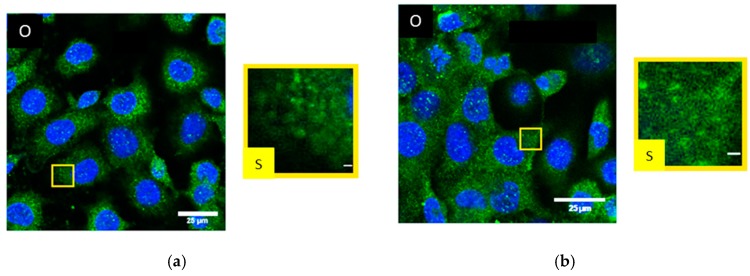
CLSM images of SkBr3 breast cancer cells after (**a**) NRG1 treatment for 1 h; (**b)** trastuzumab treatment for 1 h; blue: Cell nucleus after Hoechst34580 staining; green: Cx43 specific antibody labelling and Alexa488 staining. (O) Overview with selected region. (S) Image section of a SkBr3 cell obtained. Scale bar 1 µm.

After NRG1 treatment for 1 h (Figure 2a) as well as after trastuzumab treatment for 1 h (Figure 2b), Cx43 was more homogeneously distributed in the cytosol and the accumulations were less pronounced. The labelling pattern could not be separated between perinuclear cytosol and periphery.

**Figure 3:**
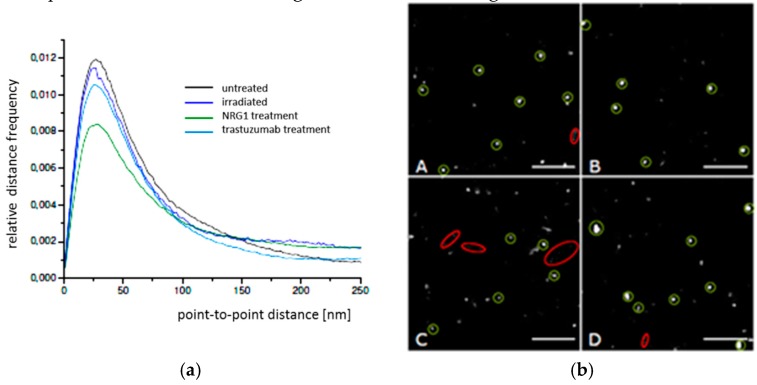
(**a**) Relative distance frequency histograms of the point-to-point distances of fluorescently labelled Cx43 molecules in SkBr3 cells for the different treatment scenarios. The distances forming the peak correlate to connexons. (**b**) Pointillist images obtained from the coordinate matrices after SMLM. The clustered points (encircled in green) represent points contributing to the peak in Figure 3a. The dispersed points (encircled in red) represent points contributing to the right “tail” of the curves in Figure 3a. A = untreated; B = irradiated; C = NRG1 treatment; D = trastuzumab treatment. Scale bar 1 µm.

By means of SMLM, frequency distributions of the point-to-point distances between each of the labelled single Cx43 molecules were calculated within the range of up to 800 nm and interpreted (Figure 3a). In all cases the relative distance frequency histograms showed a peak at approx. 27 nm distances. This distance was assumed to represent the hexamer arrangement of the connexons. The decrease of this peak for NRG1 treatment was accompanied by an increase of the frequencies of the larger distances. In Figure 3b, the results of the distance frequency distributions are represented in typical images. The distances representing the peak correlate to connexons (clustered points) whereas the dispersed points contribute to the larger distances at the right “tails” of the curves.

### 2.2. Primary Human Internal Mammary Artery Endothelial Cells (HIMAECs)

**Figure 4:**
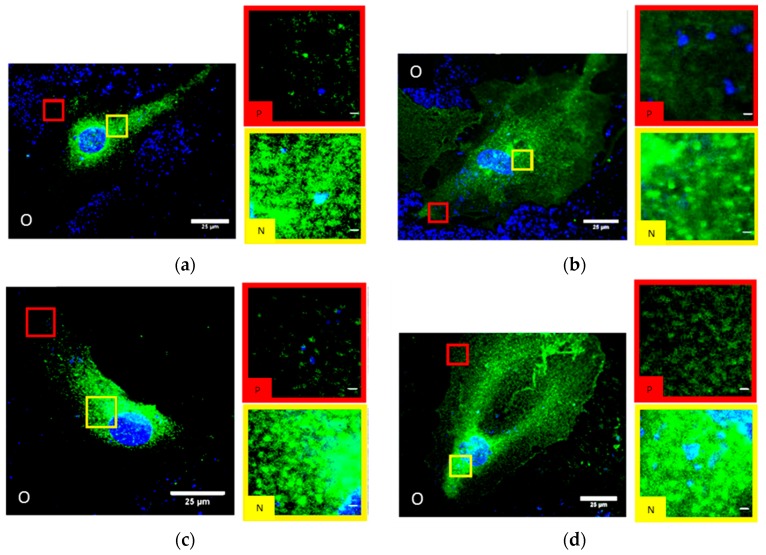
CLSM images of HIMAECs (**a**) untreated; (**b**) after 4 Gy irradiation and 50 min recovery; (**c**) after 1-h NRG1 treatment; (**d**) after 1-h trastuzumab treatment; blue: Cell nucleus after Hoechst34580 staining; green: Cx43 specific antibody labelling and Alexa488 staining. (O) Overview with selected peripheral and perinuclear regions. (P) Image section of the periphery of the cell. (N) Image section of the perinuclear region of the cell. Scale bar 1 µm.

In untreated HIMAECs, Cx43 is inhomogeneously dispersed all over the cell with ostensible enrichment near the nucleus (Figure 4a). Interpreting the CLSM images, it has, however, to be considered that HIMAECs are very flat so that the cytosol thickness near the cell nucleus is higher than the cytosol thickness in the cell periphery. The influence of cytosol thickness on the amount of Cx43 protein accessible to microscopy recording will be further discussed in chapter3. After 4 Gy irradiation and 50 min recovery, the Cx43 molecules condense to clusters and plaques (Figure 4b). After NRG1 treatment, Cx43 seems to concentrate around the cell nucleus (Figure 4c). Trastuzumab treatment also results in some accumulation around the cell nucleus but most Cx43 molecules were found in the periphery (Figure 4d).

**Figure 5:**
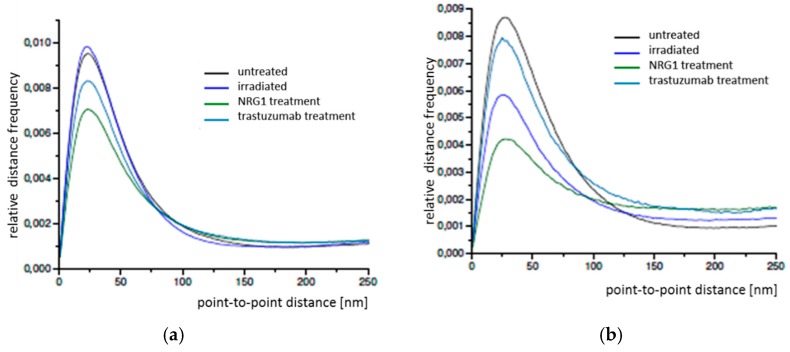
Relative distance frequency histograms of the next neighbor distances of fluorescently labelled Cx43 molecules in HIMAECs for the different treatment scenarios. The distances forming the peak correlate to connexons. (**a**) peripheral regions (**b**) perinuclear regions.

In order to better quantify potential differences in the Cx43 distributions in HIMAECs, point-to-point distances between each of the labelled single Cx43 molecules were measured by SMLM and frequency histograms of these distances were calculated for the peripheral and perinuclear regions (Figure 5). 

In all cases the relative distance frequency histograms showed a peak at about 24 nm for the peripheral regions and about 28 nm for the perinuclear regions. In perinuclear region, for all cases the peaks were lower for the treated cells in comparison to the untreated ones. The reduction in the periphery only detected in the cases of NRG1 and trastuzumab treatment, was not associated with an increase of the frequencies of the larger distances indicating a loss of Cx43 and presumably gap junctions in the membrane. In contrast, the frequencies of larger distances were increasing with the decreasing peak for the perinuclear regions indicating an increased transfer from organized Cx43 (presumably in connexons) to randomly distributed Cx43 molecules.

**Figure 6:**
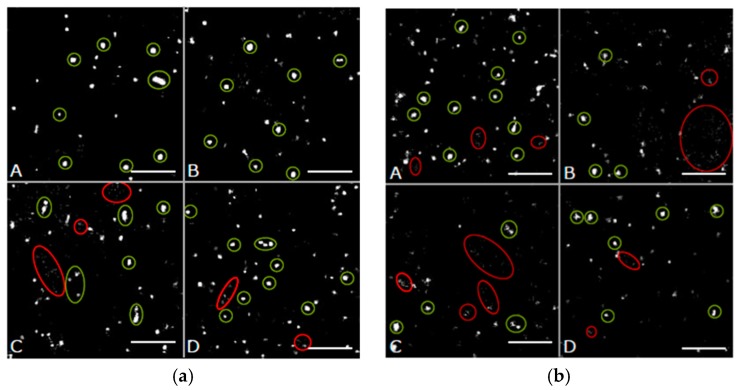
Images of Cx43 molecules in HIMAECs obtained from the coordinate matrices after Single Molecule Localization Microscopy (SMLM). The clustered points (encircled in green) represent points contributing to the peak in Figure 5. The dispersed points (encircled in red) represent points contributing to the right “tail” of the curves in Figure 5. (**a**) peripheral regions, (**b**) perinuclear regions. A = untreated; B = irradiated; C = NRG1 treatment; D = trastuzumab treatment. Scale bar 1 µm.

In Figure 6, the results of the distance frequency distributions are represented in typical images. The distances representing the peak correlate to connexons (clustered points) whereas the dispersed points contribute to the larger distances at the right “tails” of the curves.

### 2.3. Dermal Fibroblasts (BJ cells)

**Figure 7:**
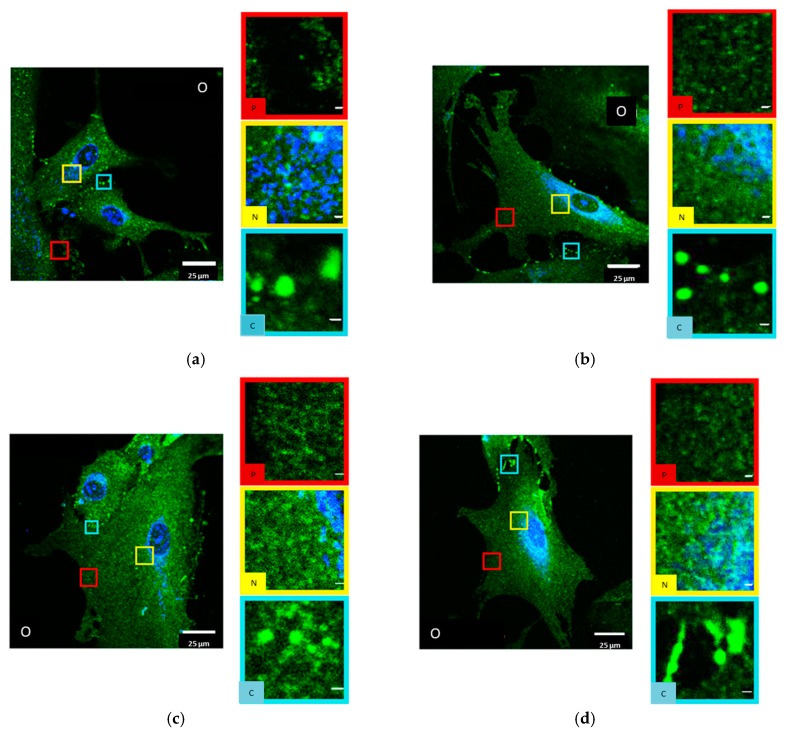
CLSM images of BJ cells (**a**) untreated; (**b**) after 4 Gy irradiation and 50 min recovery; (**c**) after 1-h NRG1 treatment; (**d**) after 1-h trastuzumab treatment; blue: Cell nucleus after Hoechst34580 staining; green: Cx43 specific antibody labelling and Alexa488 staining. (O) Overview with selected regions. (P) Image section of the periphery of the cell. (N) Image section of the perinuclear region of the cell. (C) Image section of the contact region of cells. Scale bar 1 µm.

In contrast to SkBr3 cells and HIMAECs, BJ cells showed considerable less Cx43 staining. In untreated BJ fibroblasts (Figure 7a), Cx43 appears to be homogeneously distributed over the whole cytosol. 

Only a few accumulations were visible in the contact regions between two cells. The same pattern was obtained after 4 Gy irradiation and 50 min recovery (Figure 7b). After NRG1 treatment for 1-h, small accumulations of Cx43 became visible dispersed over the whole cytosol (Figure 7c). Accumulation in the contact regions were found again. Trastuzumab treatment resulted in regions of high and low intensity especially nearby the cell nucleus (Figure 7d).

**Figure 8:**
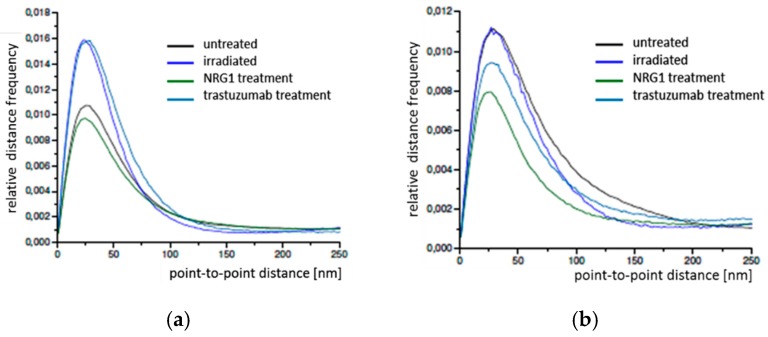

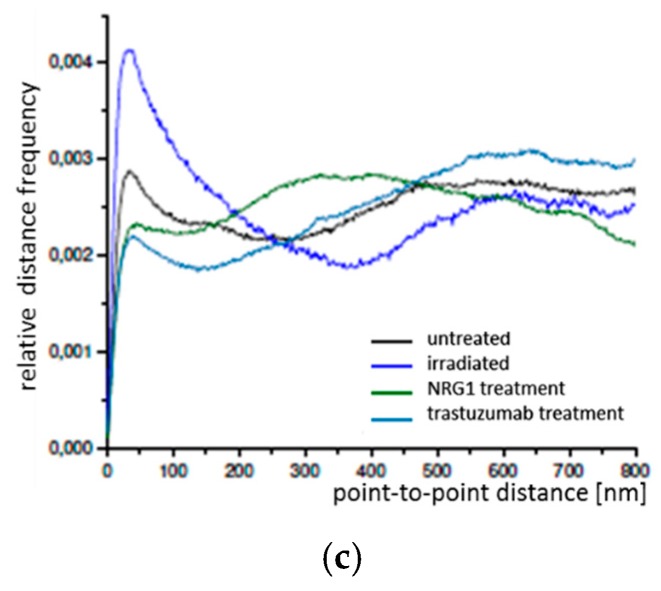
Relative distance frequency histograms of the next neighbor distances of fluorescently labelled Cx43 molecules in BJ cells for the different treatment scenarios. The distances forming the peaks in (**a**) and (**b**) correlate to connexons. (**a**) peripheral regions; (**b**) perinuclear regions; (**c**) Cx43 accumulations at the cell border. Note the different abscissa in (**c**).

In order to further quantify potential differences in the Cx43 distributions in BJ cells, point-to-point distances between each of the labelled single Cx43 molecules were measured by SMLM and frequency histograms of these distances were calculated for the peripheral and perinuclear regions (Figure 8a,b). In all cases the relative distance frequency histograms showed a peak at about 26 nm for the peripheral regions and about 28 nm for the perinuclear regions.

In contrast to the other cell types, as compared to the untreated cells a considerable increase of the peak was obtained for radiation and trastuzumab treatment for the peripheral regions indicating an increase in gab junctions as treatment response. NRG1 revealed a decrease of the peak. This behavior was not shown for the perinuclear regions where the peak maxima for untreated and irradiated cells were equal; thereby trastuzumab and NRG1 treatment revealed a peak reduction. 

Spatial distance frequency histograms were obtained for the accumulations in the contact regions of two cells (Figure 8c). Beyond the small peak at about 28–30 nm, a broad peak in the range of 250 nm to 750 nm was observed. This represents the size of the accumulations indicating an increased Cx43 density at the border of the accumulation side.

**Figure 9:**
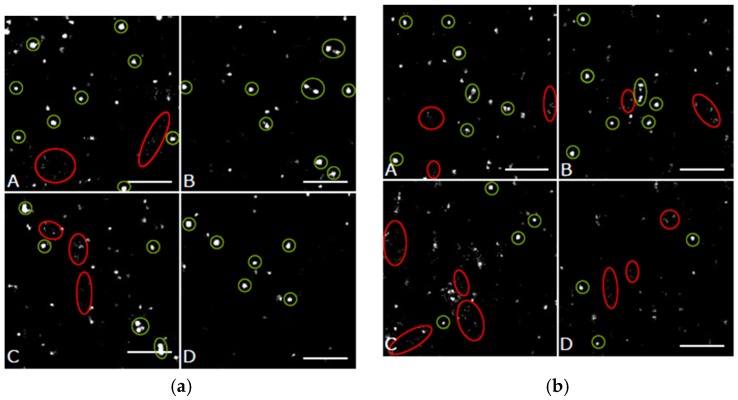
Images of Cx43 molecules in BJ cells obtained from the coordinate matrices after SMLM. The clustered points (encircled in green) represent points contributing to the peak in Figure 8a,b. The dispersed points (encircled in red) represent points contributing to the right “tail” of the curves in Figure 8a,b. (**a**) peripheral regions, (**b**) perinuclear regions. A = untreated; B = irradiated; C = NRG1 treatment; D = trastuzumab treatment. Scale bar 1 µm.

**Figure 10:**
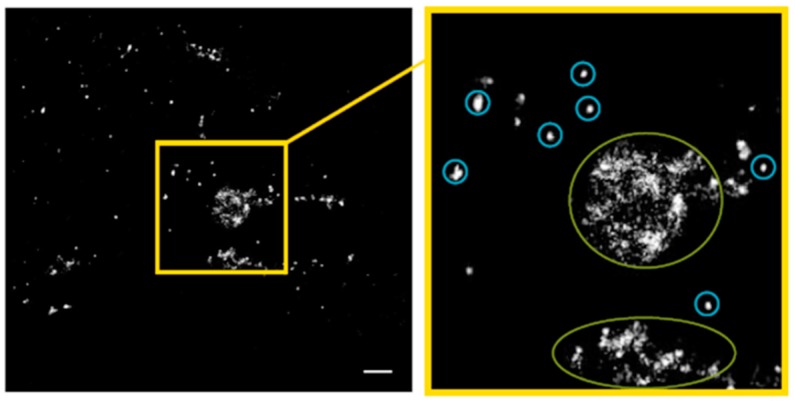
Pointillist image of Cx43 molecules in the accumulation regions at the contact zones of untreated BJ cells obtained from the coordinate matrices after SMLM. The clustered points (encircled in blue) represent points contributing to the small left peak at about 29 nm in Figure 8c. The dispersed points (encircled in green) represent points contributing to the right broad peak (>400 nm) of the curve in Figure 8c. Scale bar 1 µm.

In Figure 9, the results of the distance frequency distributions for the periphery and the perinuclear regions are represented in typical images. The distances representing the peaks correlate to connexons (clustered points) whereas the dispersed points contribute to the larger distances at the right “tails” of the curves. In contrast to the other cell types dispersed Cx43 seems to be very frequent. In Figure 10, a typical example for the regions of accumulation is shown. On one hand intact connexons contribute to the peak at about 28–30 nm (blue encircled), on the other hand the distance distribution is dominated by the large accumulation with irregular clusters inside resulting in a broad peak at distances larger than 250 nm.

## 3. Discussion

In experiments using indirect immune fluorescence for spatial identification of a particular protein like Cx43, the resulting image or coordinate based protein distribution is a superposition of several phenomena. Such phenomena can be classified as physiological ones on one hand and imaging dependent on the other. In most cases, a specific antibody does not bind to a certain protein but to a certain linear (not branched, folded or knotted) amino acid primary sequence of between 10 and 15 amino acids in length in optimum case. If not specifically designed for amino acid side chain modification like phosphorylation or glycosylation, an antibody staining therefore cannot discriminate between different modes of function of the protein identified.

In case of Cx43, this means that immune-staining and imaging via conventional non-super resolution light microscopy all states of Cx43 (in state of endoplasmic reticular located synthesis, cross cytosol antegrade or retrograde transport, or connexon associated membrane channel state) are covered by indirect immune-staining [64,65]. For Cx43 such states are reported [66]. More, the conditional state of Cx43 is reported to be under control of neuregulin-1β in cardiac myocytes, thus a similar situation in the experiments presented here must be regarded [67]. In addition, connexins not organized in gap junctions address physiological functions [68], and the functional capability of connexins is rather unspecific in that gap junction introduced as xenografts can be of functionality in the non-canonical acceptor cell [69].

The high power of localization determination of super resolution microscopy as the SMLM used in this approach is very helpful in discriminate Cx43 without being in use in gap junctions from stored but not used Cx43. The organization in gap junctions introduces a characteristic distance pattern repetitively occurring inside the hexamer channel and superposes the local aggregation field of channel hexamers characteristic for active Cx43 [30]. Cx43 not in use is randomly distributed in the cytosol or accumulated in perinuclear compartments and will show either a noisy, i.e., random, distance distribution in the plots presented (diagonal line) or will induce a peak shift as compared to plasma membrane organized ones. In brief, amount, extension and maximum peak position of the plotted Cx43 distribution identifies a peak related to Cx43 as a population with functionality and can be discriminated from dysfunctional randomly distributed Cx43 proteins displayed in the right-hand areas in the plots.

Cx43 one of the most prominent members of the 21 connexin iso-forms [31] was specifically labelled and analyzed by CLSM and SMLM [30,53,54]. In a first approach, a model system was designed consisting of (a) SkBr3 cells as a drug-sensitive breast cancer cell line with reduced Cx43 expression and increased Cx43 plaques formation [27], (b) primary human internal mammary artery endothelial cells (HIMAECs) relevant for angiogenesis, (c) BJ fibroblasts representing connective tissue in the tumor environment. These cell types were subjected to neuregulin1 (NRG1) stimulation, radiation or trastuzumab treatment. NRG1 is released in the tumor endothelium and may have a tumor protecting effect. Thus, NRG1 works in an antagonistic fashion to trastuzumab and radiation treatment.

The reduction of connexon formation induced by NRG1 in SkBr3 cells supports the tumor protective effect since the loss of GJIC is known as a typical factor towards tumor stabilization [47,48]. Furthermore, the SkBr3 data show little or even no impact on the Cx43 distribution by radiation treatment supporting the radio-resistance known for SkBr3. On the other hand, SkBr3 seems to maintain some GJIC, i.e., connexons were also observed in the periphery, which makes them accessible for drugs and thus drug-sensitive.

In HIMAECs the treatment response was different in the periphery and the perinuclear region. The SMLM data suggest strong accumulation of free Cx43 and a loss of connexons in the perinuclear regions after the different types of treatment, especially for NRG1. This effect was less pronounced in the periphery where radiation treatment seems to have no effect in comparison to the untreated cells. However, it should be considered that the irradiation experiments were accompanied by a cell recovery of 50 min that may restore damages in Cx43 distributions since connexins in general have a short half-life time and thus a high turn-over rate [32,33]. This means that the free Cx43 in the perinuclear region may also be due to the repair recycling of peripheral Cx43 in the cytosol.

It has to be considered that the cytosol thickness is a key factor for the absolute amount of Cx43 proteins, being targeted by the antibody staining. In case of endothelial, like HIMAECs, cells with tendency to maximize surface coverage on basis of a limited cytosol volume very large lateral cell body extension with very little axial thickness will result. A wide field optical recording in this case will convolute the absolute protein concentration with the recorded local cytosol thickness. This would make an estimation of protein amount by fluorescence signal intensity problematic. In CLSM recording, the axial discrimination power of a pinhole based flying spot scanner (the one used by us) is reducing this phenomenon by restricting the optical slice thickness to a value smaller than cytosol thickness in cell periphery. In brief, in the confocal case despite of cytosol thickness, variations in the local cytosol volumes compared for Cx43 amount are of the same axial extension. Therefore, even in case of very thin cells with substantial variations in cytosol thickness the fluorescence based protein amounts of different lateral cell locations can be compared.

Despite the fact that the super-resolution microscope setup is a non-confocal microscope with wide field image acquisition, the optical slicing principle can be assumed also in this case. The illumination power necessary to excite non-canonical fluorescence emission bursts in spite of canonical fluorescence traces is only reached very close to the specimen focus lateral plane. Out of focus illumination intensity shows an exponentially decreasing characteristic. This effect results in an apparent optical slicing: The stained Cx43 protein molecules do not develop fluorescence emission bursts if not suited along the lateral focus plane. Due to the high illumination intensity required and the following pronounced slope of the exponential intensity decay function the optical slice bright enough for fluorescence burst is strongly restricted in axial dimension. As a consequence, also the super-resolution recordings in spite of being recorded by wide field optics represent an optical slice across the cytosol of even thin endothelial HIMAEC cells. In both cases, confocal and super-resolution microscopy, the recorded fluorescence values per local volume are not superposed by variance in cytosol thickness.

For BJ fibroblasts the special shape of the cells leads to protrusions and contact regions to other cells. In these regions strong accumulations were observed consisting more of randomly distributed, not organized Cx43 and irregular Cx43 clusters than connexons. Radiation treatment seems to stimulate the formation of connexons in the periphery and to recruit Cx43 from the accumulation regions towards connexon formation.

So far the data presented here, were obtained from separate cell cultures. However, in tumor tissue the treatment may be accompanied by cell to cell interactions especially in those cases where an upregulation of gap junction formation occurs. Therefore future investigations will promote model systems with co-culture experiments of tumor relevant cell types. Nevertheless the approach presented here, offers new perspectives to analyze Cx43 microscopically on the single molecule level in 3D conserved cells giving new insights into the spatial organization and interaction of Cx43 in gap junctions and non-junctional cytosol.

## 4. Materials and Methods

### 4.1. Cell Culture, Treatment and Specimen Preparation

Human Internal Mammary Artery Endothelial Cells (HIMAECs; Angio-Proteomie cAP-0018) isolated from normal human internal mammary artery, were delivered at passage 3 in frozen vessels by Angio-Proteomie, Boston, MA, USA. They were cultured at 37 °C (humidified atmosphere with 5% CO_2_) in 450 mL Endothelial Basal Medium (cAP-03, Angio-Proteomie) with 2 mM L-glutamine and 50 mL Endothelial Growth Supplement (cAP-04, Angio-Proteomie) containing 10% fetal calf serum, growth factors, penicillin, and streptomycin. The cells were cultured in T-75 flasks with surfaces treated with 4 mL Quick Coating Solution (cAP-01, Angio-Proteomie) for 5 min at room temperature. For further treatment and microscopy the cells were trypsinised and transferred to coverslips positioned in 6 well plates also treated with 1 mL of the Quick Coating Solution.

The human dermal fibroblast cell line BJ (ATCC CRL-2522) was obtained commercially from the American Type Culture Collection (ATCC, Manassas, VA, USA). The cells were cultured at 37 °C (humified atmosphere with 5% CO_2_) in 500 mL Dulbecco’s Modified Eagle Medium (DMEM, Gibco 21885-025, Thermo Fisher Scientific, Waltham, MA, USA) with 2 mM L-glutamine (Gibco 25030-24), 25 mM HEPES (pH 7.4; 9105.2, Carl Roth, Karlsruhe, Germany), penicillin-streptomycin (1:100: 100 units/mL penicillin and 100 µg/mL streptomycin; Gibco 15140-122), and 10% fetal calf serum (S0615,Biochrom, Berlin, Germany). The cells were cultured without surface treatment. For further treatment and microscopy the cells were trypsinised and transferred either to coverslips positioned in 6 well plates with fresh medium.

The Her2+ breast cancer cell line SkBr3 (ATCC HTB-30) was commercially obtained from ATCC. The cells were cultured at 37 °C (humified atmosphere with 5% CO_2_) in 500 mL McCoy’s 5A (modified) medium (Gibco 36600-021), with 2 mM L-glutamine (Gibco 25030-24, Thermo Fisher Scientific), 25 mM HEPES (pH 7.4; 9105.2, Carl Roth), penicillin-streptomycin (1:100; Gibco 15140-122), and 10% fetal calf serum (Biochrom S0615). The cells were growing without surface treatment. For further treatment and microscopy the cells were trypsinised and transferred to coverslips positioned in 6 well plates with fresh medium. Aliquots of each sample were additionally treated as follows:

(a) Irradiation: Cells were irradiated with the linear accelerator ARTISTE (Siemens, Erlangen, Germany), using 6 MeV photon energy at a radiation dose of 4 Gy (dose rate 3 Gy/min). After 50 min the cells were fixed.

(b) Neuregulin1 (NRG1) treatment: 5 h before application of NRG1, the medium was exchanged by the same medium but with 0.5% fetal calf serum only instead of 10%. The cells were treated with 100 ng/mL NRG1 (3–4 mL/well; ab50227, Abcam, Cambridge, UK), for 1 h and fixed as described below. This NRG1 concentration is about 200 times the ED_50_-value according to the product data sheet.

(c) Trastuzumab treatment: For the treatment with trastuzumab (Herceptin^®^, kindly provided by Roche, Penzberg, Germany), a serum reduction as described for NRG1 took place. Afterwards, cells were treated with medium without fetal calf serum containing 172 nM trastuzumab for 1 h and then fixed as described below.

Before microscopy, the cells were fixed. The medium was removed and the cells were washed with 3–4 mL prewarmed 1× PBS (phosphate buffered saline) with 1 mM Ca^2+^ and 2 mM Mg^2+^ and incubated twice in 6% formaldehyde (prepared freshly from 37% formaldehyde (7040, J.T. Baker Analytics, Munich, Germany)) for 20 min at 37 °C each. The formaldehyde was removed by washing the cells 5 times in 1× PBS for 5 min each. Finally, the cells were stored in 1× PBS and 0.1% NaN_3_ (0326, J.T. Baker Analytics) at 4 °C until further use.

### 4.2. Labelling and Microscopy

The cell nuclei were stained with Hoechst34580 (1 µg/mL) for 10 min and washed 5 times in 1× PBS for 5 min each. Cx43 was labelled with a primary polyclonal rabbit anti-Connexin43/GJA1 antibody (750 ng/mL; ab11370, Abcam) for 18 h at 4 °C. After washing 8times in 1× PBS for 4 min each, the secondary donkey anti-rabbit Alexa-488 antibody (2 µg/mL; ab150061, Abcam) or Alexa-647 antibody (2 µg/mL; ab150061, Abcam) was added for CLSM or SMLM, respectively, and the cells were incubated and washed as for the primary antibody.

Confocal laser scanning microscopy was performed with a Leica TCS SP5 (Leica Microsystems, Wetzlar, Germany). The specimens were illuminated at 405 nm, 561 nm, or 633 nm laser wave length respectively. The emission signal was collected by an oil immersion objective HCX PL APO 63×/NA 1.32 at 414–463 nm (cell nuclei Hoechst34580 staining), at 570–623 nm (Cx43 Alexa-488 labelling), or at 642–732 nm (Cx43 Alexa-647 staining), respectively. 2D image sections of 246 µm × 246 µm (2048 × 2048 pixels) were acquired.

SMLM was performed using a specially manufactured localization microscope described in detail elsewhere [58,59,70]. The optical setup comprises enhanced thermomechanical stabilization, indoor climate regulation and separate cooling of optical elements so that the temperature of the setup was maintained in the range of ± 10 mK. The laser beam was expanded to a circular profile, flattened and projected into the object plane using a 100×/NA 1.46 oil plan apochromatic objective lens (Carl Zeiss Microscopy, Göttingen, Germany). For the AlexaFluor647 dye used here, the illumination wavelength of 642 nm with about 1 kW/cm^2^ power density in the object plane was used. The fluorescence light was separated from the illumination light using appropriate filters and was recorded by the Andor Ultra EMCCD camera (Andor Technology, Belfast, Northern Ireland). All images were acquired after a 1 h start-up phase for thermal stabilization. For each cell nucleus, a time stack of 3000 image frames with an integration time of 100 ms each was registered and saved in 16-bit grey-scale TIFF image stack format. For each specimen 10 up to 20 cells were recorded and evaluated.

For data analysis of SMLM data from the raw images in-house built MATLAB-based programs were applied as described in detail elsewhere [30,54,58,59,63,71,72]. The local positions of the dye molecules were detected from the blinking events and a data matrix was produced which contains information about signal amplitude, the lateral x- and y-coordinates, the standard deviations in x and y direction, position errors, etc. From this matrix, point-to-point distances between 0 and 800 nm were calculated and distance frequency histograms derived. Additionally, pointillist images of the loci were reconstructed.

## 5. Conclusions

Cx43 is one of the prominent connexins in the formation of gap junctions. Studies in cancer cells have suggested that also non-junction activities of Cx43 are important for tumor progression and the formation of metastasis. Understanding mechanisms behind Cx43 upregulation, gap junction formation, and non-junction activities might help to improve chemotherapy and tumor treatment. This requires the analysis of model systems containing tumor cells and tumor environmental cell types. We have presented such a model system and have applied radiation, NRG1, and trastuzumab treatment to this system. Using SMLM, we have demonstrated new approaches to quantify Cx43 topologies on the nano-scale and to investigate cell regions of interest as detected by CLSM. The data presented here allow a detailed description of the formation of connexons and enrichment of free Cx43 in different cell regions. This novel methodological approach offers new insights into Cx43 trafficking in tumor related cells on a single molecule scale under different treatment conditions.
